# *SPI1* is a prognostic biomarker of immune infiltration and immunotherapy efficacy in clear cell renal cell carcinoma

**DOI:** 10.1007/s12672-022-00592-0

**Published:** 2022-12-07

**Authors:** Huayi Feng, Tao Wang, Jiali Ye, Yang Yang, Xing Huang, Dong Lai, Zheng Lv, Yan Huang, Xu Zhang

**Affiliations:** 1 Medical School of Chinese PLA, Beijing, 100853 China; 2 Department of Urology, The Third Medical Center, Chinese PLA General Hospital, Beijing, 100853 China; 3 Department of Urology, The Tianjin Third Central Hospital Affiliated of Nankai University, Tianjin, China

**Keywords:** *SPI1*, Clear cell renal cell carcinoma, Prognosis, Immune infiltration, Methylation, Immunotherapy

## Abstract

**Background:**

Spi-1 proto-oncogene (*SPI1*), which encodes an ETS-domain transcription factor, can activate gene expression in myeloid and lymphoid lineages. The role of *SPI1* in the tumor immune microenvironment in clear cell renal cell carcinoma (ccRCC) remains unknown. In this study, we investigated the possible role of *SPI1* in ccRCC using an independent cohort and a comprehensive bioinformatics analysis.

**Materials and methods:**

Quantitative real-time PCR, western blot and immunohistochemistry assays were used to compare the *SPI1* expression levels between ccRCC tissues and normal tissues, analyze the relationships between *SPI1* and *CD68, CD8, CD4* expression levels, and explore the link between *SPI1* and the efficacy of immunotherapy in our cohort. Tumor Immune Estimation Resource, UALCAN, cBioPortal, TISIDB database, and LinkedOmics database were used in our study.

**Results:**

*SPI1* expression level was higher in ccRCC bulk tissues than in normal bulk tissues. *SPI1* was an independent prognostic factor for poor overall survival and progression-free survival in patients with ccRCC. *SPI1* expression was strongly related to the infiltration of immune cells and immune-related molecules. *SPI1* was more highly expressed in tumor-infiltrating immune cells rather than in cancer cells. Non-responders to immunotherapy against ccRCC were more likely to express higher *SPI1* levels than responders. Genes co-expressed with *SPI1* primarily correlated with immune-related pathways.

**Conclusions:**

*SPI1* expression in tumor bulk tissues is associated with disease progression and poor prognosis, as well as high expression levels of immune markers and infiltration of immune cells. *SPI1* can be used as a prognostic biomarker to monitor and evaluate immunotherapy efficacy.

**Supplementary Information:**

The online version contains supplementary material available at 10.1007/s12672-022-00592-0.

## Introduction

Clear cell renal cell carcinoma (ccRCC), a tumor with high immune infiltration, is a major histological subtype of renal cell carcinoma [1]. Although cancer treatment, particularly cancer immunotherapy, has greatly advanced in recent years, patients with metastatic and advanced ccRCC have poor prognoses [2]. Additionally, effective markers for monitoring and evaluating immunotherapy efficacy are lacking.

The tumor immune microenvironment (TME), which contains tumor cells, immune cells, their surrounding stroma, and extracellular components, affects tumor progression and immunotherapy efficacy [3]. Tumor progression or suppression is most dependent on the immunosuppressive or immunostimulatory state [4]. T-cell exhaustion and tumor-infiltrating lymphocytes (TILs) are associated with an exhausted phenotype and limited anti-tumor activity [5]. Tumor-associated macrophages, myeloid-derived suppressor cells (MDSCs), Treg cells, and neutrophils are known to promote tumor progression by creating an immunosuppressive tumor environment. Modulated by cytokines and chemokines, tumor-infiltrating immune cells (TIICs) interact with cancer cells in an extensive and dynamic manner and play essential roles in TME [6]. TIICs not only affect tumor development, progression, and response to immunotherapy, but also predict clinical outcomes in patients with cancer [6]. Although several molecules (PD-1, CTLA4, LAG3, TIM3, and TIGIT) in immune cells may be useful markers for prognosis and immunotherapy efficacy in cancer [7], more reliable and effective biomarkers for individualized prognosis and therapy selection are urgently needed.

Spi-1 proto-oncogene (*SPI1*) encodes PU.1 (a member of the E26-transformation-specific transcription factor family), an essential factor in signal transmission in the immune system and the development of myeloid cells and lymphocytes [8]. Macrophage differentiation is regulated by *SPI1*-related gene sets [9]. In addition, *SPI1* is important in malignant diseases. *SPI1* has been identified to facilitate glycolytic processes and promote colon cancer progression [10]. The *SPI1*-METTL14-MYB/MYC signaling axis participates in myelopoiesis and leukemogenesis [11]. Transcriptional activation of *SPI1* can enhance the tumorigenic potential of cervical cancer cells through its effect on PARP9 [12]. Importantly, Li et al. demonstrated that the transcription factor *SPI1* can promote the expression of CD86, CCL4/CXCL10/CX3CL1, and MHC-II, thereby increasing immune infiltration [13]. However, the relationship between of *SPI1* expression and immune cell infiltration in ccRCC remains unclear.

In our cohort, *SPI1* was significantly overexpressed in ccRCC stroma and was closely related to several clinicopathological parameters of ccRCC, including the Furman grade, T stage, nodal metastasis, and metastasis. In ccRCC bulk tissues, DNA methylation levels of *SPI1* was lower than that in normal bulk tissues. Furthermore, *SPI1* was found to be an independent indicator of poor OS and PFS in patients with ccRCC. Interestingly, immunohistochemistry revealed that *SPI1* was more highly expressed in TIICs rather than in cancer cells, consistent with the SPI1 expression in renal cell lines. *SPI1* expression and methylation status were strongly correlated with immune cell infiltration and immune-related molecules in ccRCC. For immunotherapy in RCC, non-responders were more likely to express higher *SPI1* levels than responders. Genes co-expressed with *SPI1* are associated with various immune processes. Thus, *SPI1* expression level may be a marker of poor prognosis and immunotherapy efficacy associated with immune cell infiltration.

## Materials and methods

### Patients and samples

A ccRCC tissue microarray (TMA) containing primary tumor tissues and paired adjacent normal tissues of 183 patients was obtained from the Department of Urology, The First Medical Center, Chinese PLA General Hospital, Beijing, China. Primary tumor tissues from seven patients undergoing immunotherapy plus targeted therapy (pembrolizumab plus axitinib) after radical nephrectomy were also paraffin-embedded and sectioned for subsequent experiments. This study was approved by the Ethics Committee of the Chinese PLA General Hospital.

### Clinical cohorts

Patient data from three prospective clinical trials of the anti-PD-1 antibody nivolumab in ccRCC, including CheckMate 009 (CM-009; NCT01358721) [14], CheckMate 010 (CM-010; NCT01354431) [15], and CheckMate 025 (CM-025; NCT01668784) [16] were used to explore the relationship between *SPI1* expression and immunotherapy efficacy in this study [17]. We obtained 129 ccRCC patients with primary tumor RNA sequencing data and complete clinical efficacy of nivolumab (Additional file 3: Table S3). Clinical benefit (CB) encompassed patients who achieved complete responses or partial responses, or stable disease with tumor shrinkage and PFS of at least 6 months. No clinical benefit (NCB) encompassed patients who achieved progressive disease and PFS less than 3 months. All other patients were classified as having intermediate clinical benefit (ICB) [17]. Patients were stratified into low or high groups based on *SPI1* expression, using the median RNA expression of *SPI1* as the cut-off value. Correlations between *SPI1* expression and clinical efficacy of nivolumab was analyzed by the Pearson’s Chi squared test.

### Quantitative real-time PCR (qRT-PCR)

qRT-PCR was used to determine *SPI1* mRNA expression levels in ccRCC and normal bulk tissues. To extract total RNA, an RNA-Quick Purification Kit (RN001; ESscience Biotech, China) was used. Complementary DNA was synthesized using a kit from BIO-RAD, iScriptTM cDNA Synthesis Kit (1708890). For quantitative PCR, iTaq Universal SYBR® Green Supermix (1725120, Bio-Rad, USA) was used. The 2-∆∆Ct method was used to analyze the relative mRNA expression level of *SPI1* normalized to the level of peptidylprolyl isomerase A (PPIA). The forward primer sequence for *SPI1*: GTGCCCTATGACACGGATCTA, reverse primer: AGTCCCAGTAATGGTCGCTAT. The process was performed with gene-specific primers and SYBR Green detection using QuantStudio 3 (Applied Biosystems).

### Western blot

Western blot assays were performed in accordance with standard techniques as previously reported [18]. Antibodies against SPI1 (66618–2-lg; Proteintech) andβ-Tubulin (BE0025; EASYBIO) were used.

### Immunohistochemistry (IHC)

IHC staining was used to examine the expression level of SPI1 protein in cancerous and paracancerous tissues from our cohort. IHC staining of SPI1 (Atlas, HPA055653), CD68 (Abcam, ab955), CD8 (Proteintech, 66868–1-Ig), and CD4 (Abcam, ab133616) was performed on TMA tissues. We followed standard protocols, as described previously [19]. The slides were scaned using a Panamic 250 Flash III slide scanner (3DHISTECH Ltd, Budapest, Hungary) and images were analyzed using CaseViewer software (3DHISTECH Ltd, Budapest, Hungary) [20]. Positive cells were counted in five random fields (400X) [21].

### Cell culture

Fetal bovine serum (FBS) and different type of media including high glucose-DMEM, MEM, RPMI-1640, and McCoy’s 5A was purchased from EVERY GREEN (Hangzhou, China) and VIVICUM bioscience (Beijing, China), respectively. The human embryonic kidney derived cell line HEK293T, human renal tubular epithelial cell line HKC and HK2, human ccRCC cell lines SN12, A498, 786O, ACHN, OS-RC-2, and Caki-1 were originally purchased from American Type Culture Collection. The murine renal carcinoma cell line RENCA were purchased from Peking Union Medical College. RAW246.7 macrophages were purchased from BeNa Culture Collection (Beijing, China). Cell culture conditions were as describe [22].

### Tumor immune estimation resource (TIMER) analysis

In the TIMER (https://cistrome.shinyapps.io/timer/) database [23], correlation modules and gene modules were used to analyze the relationship between *SPI1* expression and gene markers of immune cells [24, 25].

### UALCAN analysis

Using the UALCAN (http://ualcan.path.uab.edu/) database [26, 27], the expression and methylation status of *SPI1* were examined in different sample types of ccRCC by Wilcoxon rank sum test.

### TISIDB database analysis

Using the TISIDB database (http://cis.hku.hk/TISIDB) [28], the associations between the methylation status of *SPI1* and infiltrations of lymphocytes, immunomodulators, chemokines, and chemokine receptors were determined.

### cBioPortal analysis

Using the cBioPortal (www.cbioportal.org) database [29], the relationship between the mRNA expression and methylation levels of *SPI1* were determined by Spearman correlation coefficient analysis. (Additional file 4: Fig. S1b).

### LinkedOmics database analysis

In the LinkedOmics (http://www.linkedomics.org/login.php) database [30], differentially expressed genes related to *SPI1* in ccRCC were identified. Correlation analysis using the Pearson correlation coefficient is shown in Additional file 2: Table S2.

### Statistical analysis

Histogram generation was performed using GraphPad Prism software (version 8.0). Scatter plots were generated using the “ggplot” (version 3.3.3) packages in R. “survival” (version 3.2–10) package and “survminer” (version 0.4.9) package in R were used to draw survival curves. Wilcoxon rank sum test was used to analyze the differential expression levels of SPI1 protein between the ccRCC tissues and the adjacent normal tissues in our cohort. Correlations between SPI1 expression and clinicopathological parameters were analyzed by the Pearson’s Chi squared test. Patients were stratified into low or high groups based on SPI1 expression, using the median numbers of SPI1 positive cells per 20000um^2^ as the cut-off value. Univariate and multivariable Cox proportional hazards regression analyses were performed to identify potential prognostic factors associated with OS and PFS using the “survival” package of R. Nomogram analysis was conducted using the “survival” (version 3.2–10) and “rms” (version 6.2–0) packages to establish the risk prediction model. The correlation between SPI1 expression and CD68, CD8, and CD4 expression in ccRCC was analyzed by Spearman correlation coefficient analysis. All statistical analyses were performed using GraphPad Prism version 8.0 software (GraphPad, Inc., La Jolla, CA, USA) and R, version 3.6.3 software (The R Group for Statistical Computing, Vienna, Austria).

## Results

### SPI1 expression in ccRCC

**Fig. 1:**
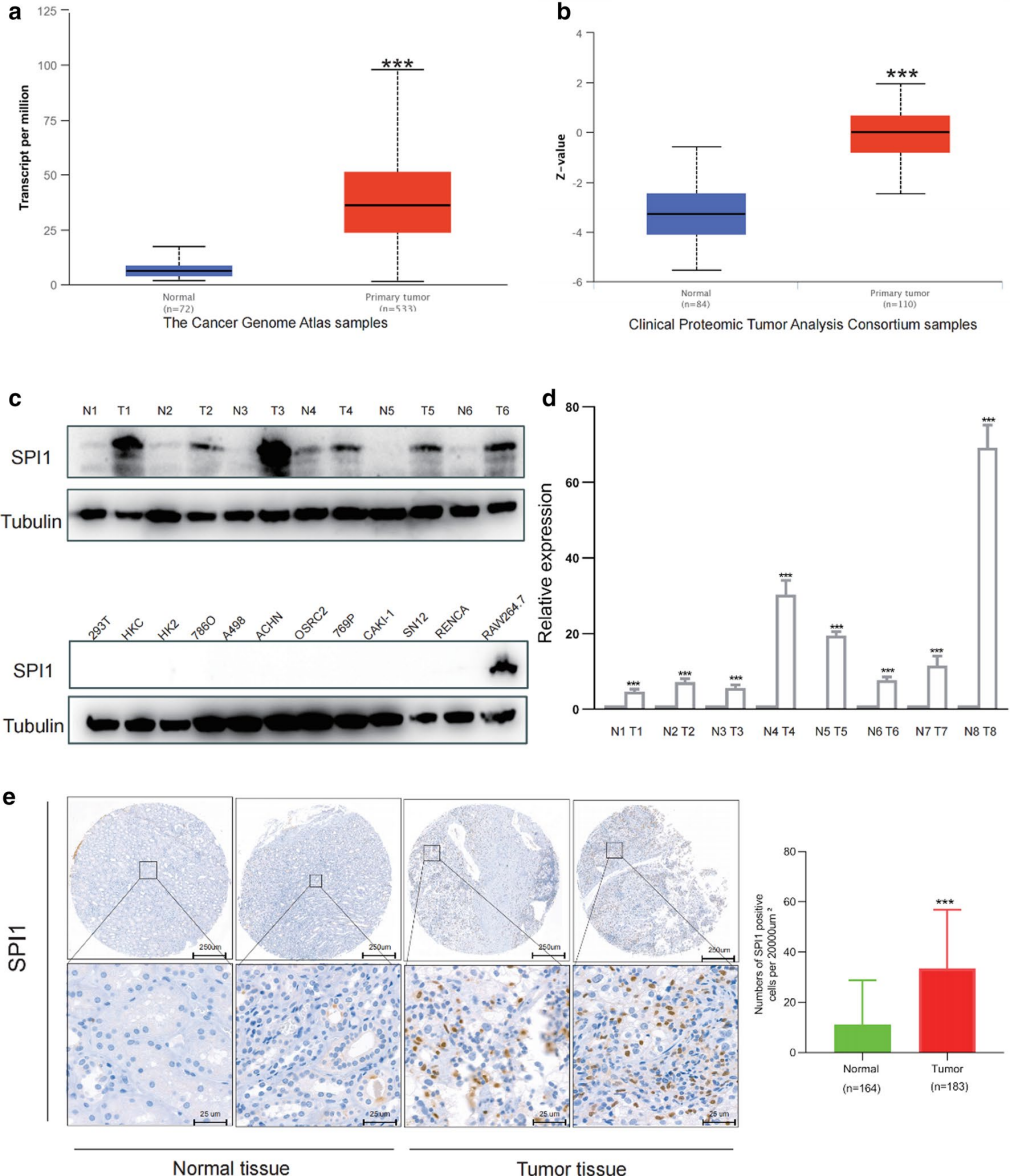
*SPI1* expression in ccRCC. **a**, **b** SPI1 protein and mRNA expression levels were elevated in ccRCC bulk tissues compared to normal bulk tissues in the UALCAN database. **c** SPI1protein expression levels in six pairs ccRCC tumor bulk tissues and adjacent normal bulk tissues as well as ccRCC cell lines. **d** SPI1 mRNA expression levels in eight ccRCC tumor bulk tissues and adjacent normal bulk tissues (**e**) *SPI1* protein expression levels was elevated in ccRCC stroma compared to normal stroma in our cohort. ccRCC, clear cell renal cell carcinoma; *, p < 0.05; **, p < 0.01; ***, p < 0.001

UALCAN database analysis revealed that SPI1 protein and mRNA expression levels were elevated in ccRCC bulk tissues compared to that in normal bulk tissues (Fig. 1a, b). Western blot assay, qRT-PCR analysis and IHC staining confirmed that SPI1 mRNA and protein expression levels were upregulated in ccRCC bulk tissues compared to those in normal bulk tissues (Fig. 1c–e). SPI1 staining was mainly observed in TIICs rather than in tumor cells (Fig. 1e). We evaluated the methylation levels of *SPI1* in the ccRCC dataset and found that tumor bulk tissues had significantly lower DNA methylation levels than the normal bulk tissues (Additional file 4: Fig. S1a). Correlation analysis showed that *SPI1* mRNA expression was negatively correlated with methylation status (Additional file 4: Fig. S1b). Furthermore, hypomethylation of *SPI1* was more likely to occur in late-stage, high-grade tumors, and those with N1 nodal metastasis status (Additional file 4: Fig. S1c–e). Based on these results, *SPI1* mRNA and protein levels were higher in ccRCC bulk tissues compared to normal bulk tissues.

### Prognostic value of SPI1 and its correlation with clinicopathological parameters in ccRCC

**Fig. 2:**
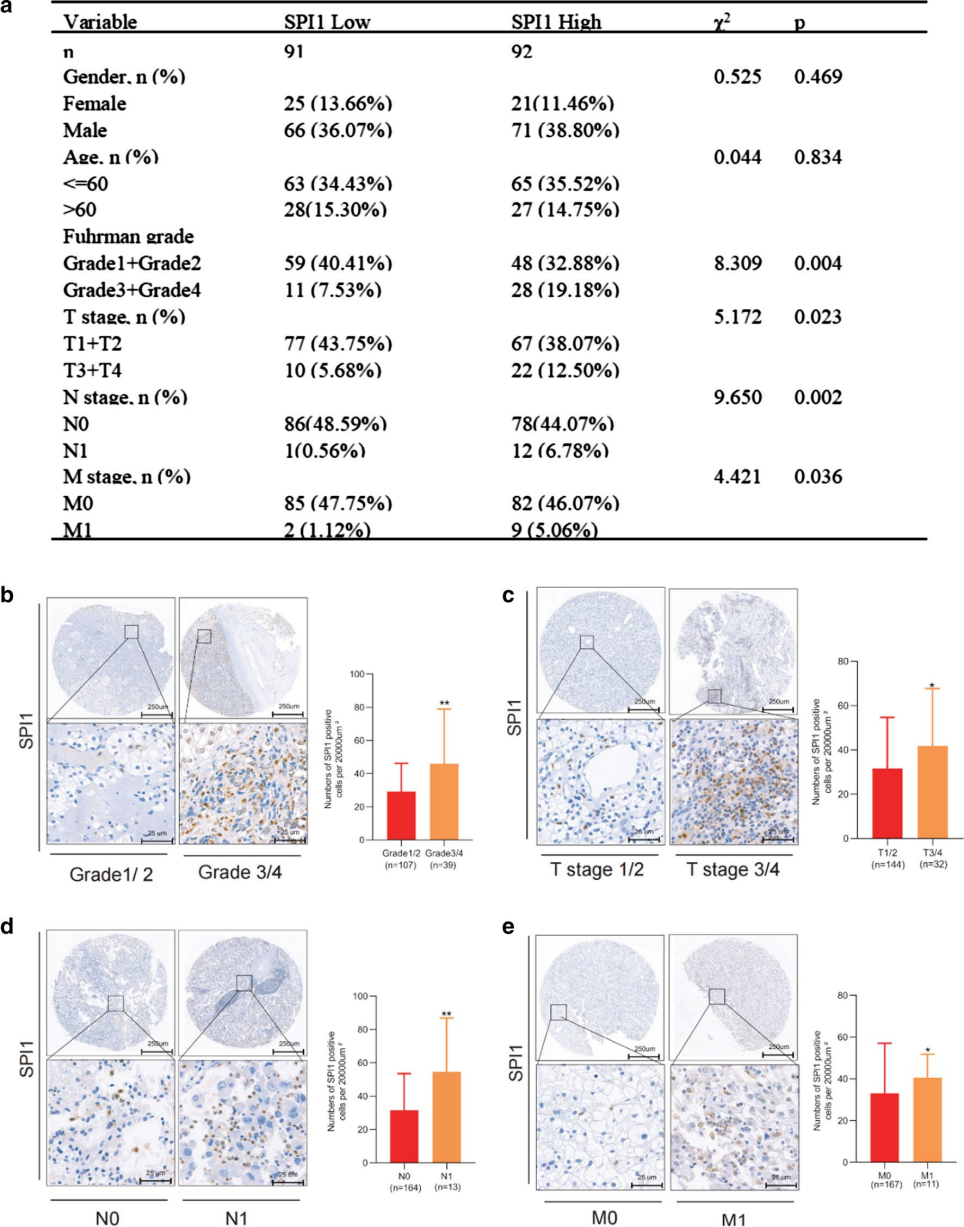
Relationship between *SPI1* expression and clinicopathological parameters in ccRCC. **a** Clinicopathological parameters correlated with *SPI1* expression. **b**–**e** High *SPI1* expression level was associated with high Furman grade (**b**), T stage (**c**), positive nodal status (**d**), and metastasis status (**e**). ccRCC, clear cell renal cell carcinoma; *, p < 0.05; **, p < 0.01; ***, p < 0.001

**Fig. 3:**
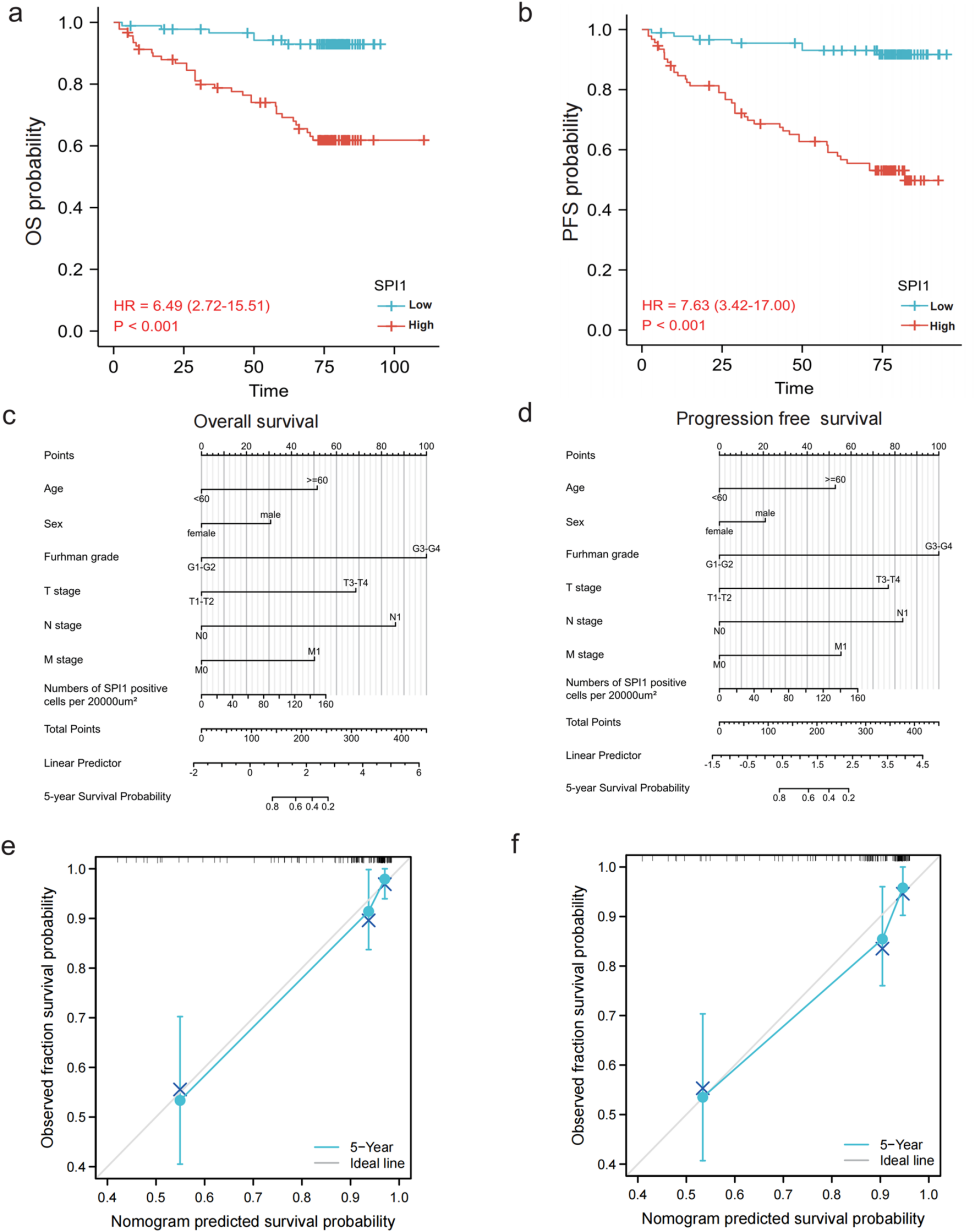
Prognostic value of *SPI1*. **a**, **b** High *SPI1* expression level predicted poor OS (**a**) and PFS **b** in ccRCC. **c**, **d** OS (**c**) and PFS (**d**) predictions of patients with ccRCC at five years after surgery are shown in nomograms. **e**, **f** Calibration plots of the nomograms for the prediction of five-year OS **e** and PFS **f**. *ccRCC* clear cell renal cell carcinoma; *OS* overall survival; *PFS* progression-free survival

The relationships between SPI1 and the clinicopathological features of patients with ccRCC were explored in our cohort with 183 ccRCC patients from the Department of Urology, The First Medical Center, Chinese PLA General Hospital, Beijing, China, and clinicopathological parameters correlated with SPI1 expression, such as Furman grade, T stage, N stage, and M stage were identified (Fig. 2a).High SPI1 expression level was associated with high Furman grade, T stage, positive nodal status, and metastatic status (Fig. 2b–e). SPI1 expression was not associated with number of lymph nodes (regional lymph node metastases involving one node vs. regional lymph node metastases involving more than one node) and the site of metastasis. (data no shown). High SPI1 expression levels predicted poor OS (hazard ratio [HR] = 6.49, p < 0.001) and PFS (hazard ratio [HR] = 7.63, p < 0.001) in patients with ccRCC (Fig. 3a, b). Multivariate Cox regression analysis indicated that SPI1 expression, age, Furman grade, T stage, and M stage were independent prognostic factors for OS in patients with ccRCC (Table 1). SPI1 expression, age, Furman grade, and T stage were independent prognostic factors for PFS in ccRCC patients (Table 2). Finally, the overall five-year survival rate and progression-free five-year survival rate were estimated using nomograms (Fig. 3c, d). Calibration plots of the nomograms for the prediction of five-year OS (Fig. 3e) and PFS (Fig. 3f) are shown. These results suggest that SPI1 is associated with a poor clinical prognosis in ccRCC.

**Table 1:** Univariate and multivariable cox regression of *SPI1* expression for overall survival in patients with ccRCC

Variable	Univariate cox regression			Multivariable cox regression		
	HR	95%CI	p-value	HR	95%CI	p-value
Age: > 60 (n = 55) vs ≤ 60 (n = 128)	2.035	1.086 ~ 3.814	0.027	2.707	1.183 ~ 6.192	0.035
Gender: male (n = 137) vs female (n = 46)	1.290	0.593 ~ 2.806	0.521	1.423	0.506 ~ 3.999	0.504
Furman grade: G3 + G4 (n = 39) vs G1 + G2 (n = 107)	10.624	4.860 ~ 23.198	0.000	7.466	3.158 ~ 17.649	0.000
T stage: T3 + T4 (n = 32) vs T1 + T2 (n = 144)	12.750	6.510 ~ 24.970	0.000	4.054	1.699 ~ 9.674	0.002
N stage: N1 (n = 13) vs N0 (n = 164)	12.777	6.001 ~ 27.202	0.000	2.597	0.873 ~ 7.721	0.086
M stage: M1 (n = 11) vs M0 (n = 167)	6.864	3.113 ~ 15.134	0.000	3.774	1.097 ~ 12.982	0.035
SPI1: high (n = 92) vs low (n = 91)	6.479	2.713 ~ 15.473	0.000	7.471	2.324 ~ 24.013	0.001

**Table 2:** Univariate and multivariable cox regression of *SPI1* expression for progression-free survival in patients with ccRCC

Variable	Univariate cox regression			Multivariable cox regression		
	HR	95%CI	p-value	HR	95%CI	p-value
Age: > 60 (n = 55) vs ≤ 60 (n = 128)	2.017	1.076 ~ 3.781	0.029	2.487	1.106 ~ 5.592	0.027
Gender: male (n = 137) vs female (n = 46)	1.279	0.588 ~ 2.783	0.535	1.525	0.552 ~ 4.219	0.416
Furman grade: G3 + G4 (n = 39) vs G1 + G2 (n = 107)	9.709	4.454 ~ 21.166	0.000	6.500	2.737 ~ 15.440	0.005
T stage: T3 + T4 (n = 32) vs T1 + T2 (n = 144)	11.713	5.990 ~ 22.907	0.000	3.338	1.436 ~ 7.762	0.002
N stage: N1 (n = 13) vs N0 (n = 164)	12.053	5.713 ~ 25.430	0.000	2.236	0.771 ~ 6.483	0.138
M stage: M1 (n = 11) vs M0 (n = 167)	5.418	2.476 ~ 11.856	0.000	3.277	0.977 ~ 10.985	0.055
SPI1: high (n = 92) vs low (n = 91)	6.718	2.811 ~ 16.052	0.000	7.422	2.349 ~ 23.454	0.001

### SPI1 is widely expressed in immune cell types correlating with immune infiltration and immunotherapy efficacy in ccRCC

**Fig. 4:**
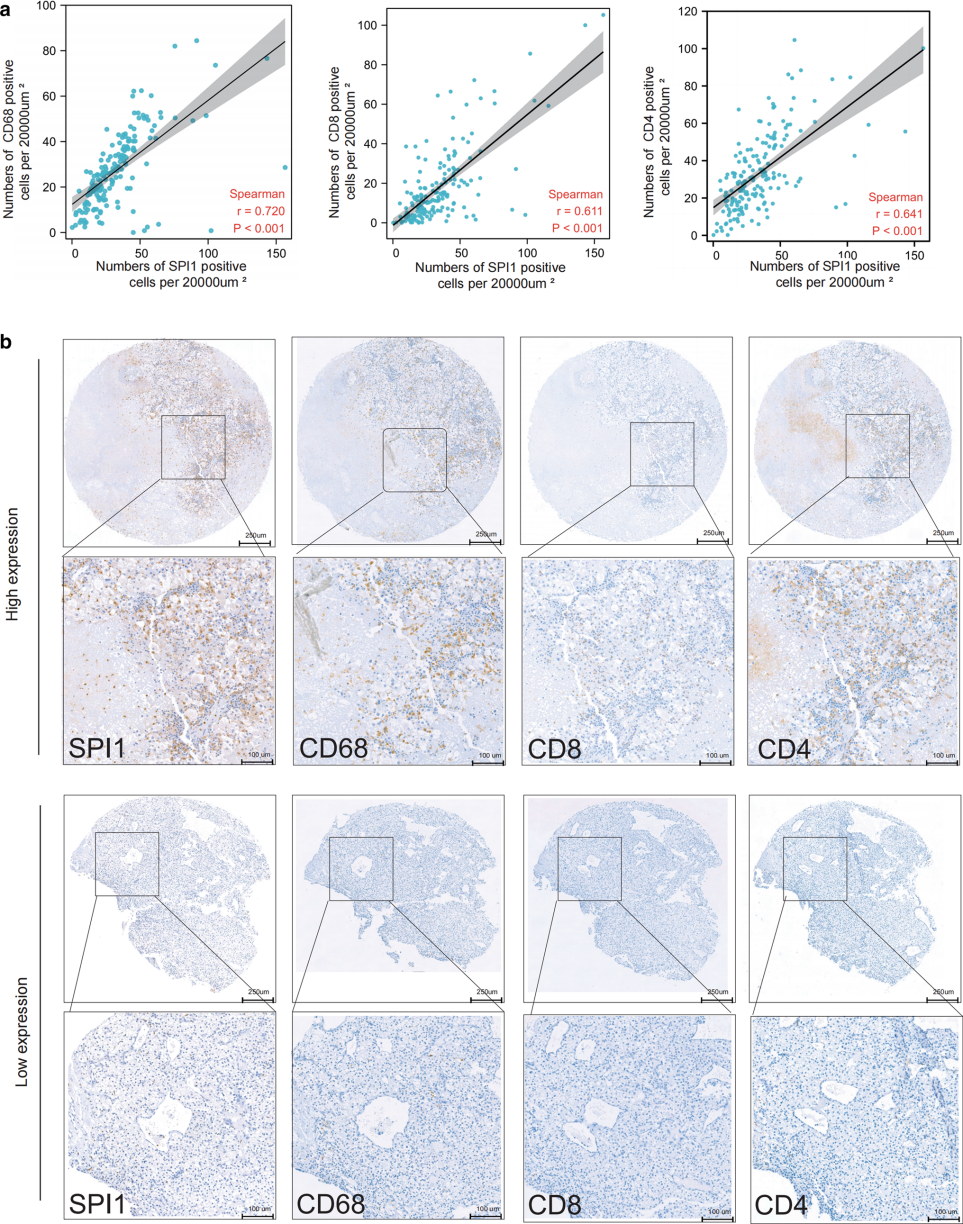
Relationship between *SPI1* expression and CD68 + macrophages, CD4 + T cells, and CD8 + T cells infiltration. **a** *SPI1* expression correlated positively with CD68 + macrophages, CD4 + T cells, and CD8 + T cells infiltration in our cohort. **b** *SPI1*, *CD68* + *, CD8* + *, and CD4* + expression levels in the same patients are shown by IHC. ccRCC, clear cell renal cell carcinoma; *, p < 0.05; **, p < 0.01; ***, p < 0.001; IHC, immunohistochemistry

**Fig. 5:**
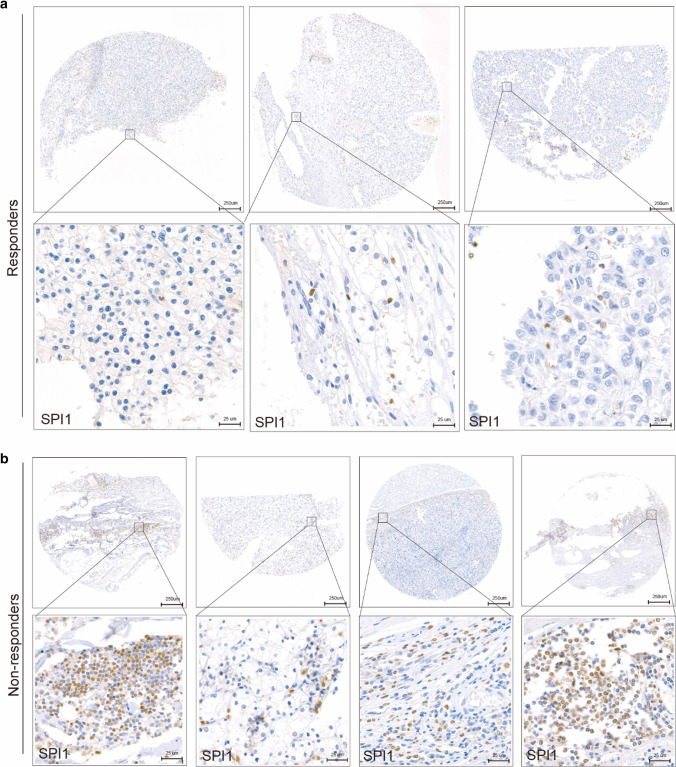
Relationship between *SPI1* expression and immunotherapy efficacy. **a** *SPI1* expression in three responders receiving immunotherapy. **b** *SPI1* expression in four non-responders receiving immunotherapy. Responders included patients with complete disease and partial disease, and non-responders included patients with progressive disease and stable disease

Tumor tissues contain large numbers of non-tumor cells, of which immune cells represent a major fraction [31]. According to the results of IHC staining, SPI1 was mainly expressed in the cell nuclei of immune cells, rather than in tumor cells, implying that SPI may be a biomarker of immune cells infiltration in TME. We evaluated the correlations between SPI1 expression and *CD68, CD8*, and *CD4* expression in ccRCC using our cohort and found that SPI1 expression level was positively correlated with infiltrating levels of CD68 + macrophages, CD4 + T cells, and CD8 + T cells (Fig. 4a). The TME with high SPI1 expression level can simultaneously maintain high macrophage, CD8 + T cell, and CD4 + T cell infiltration (Fig. 4b). To further explore the relationship between *SPI1* and immunity in ccRCC, we performed a correlation analysis adjusted for tumor purity between *SPI1* and gene markers of immune cells and stromal cells using the TIMER platform. *SPI1* was strongly correlated with gene markers of CD8 + T cells, Tregs, monocytes, tumor-associated macrophages, M2 macrophages, neutrophils, dendritic cells, regulatory T cells (Tregs), and T cell exhaustion (Table 3). Thus, *SPI1* can be a biomarker of immune infiltration in the TME of ccRCC. To investigate the association between SPI1 expression and immunotherapy efficacy in RCC, IHC staining of SPI1 was performed in six patients with RCC who had received at least four cycles of pembrolizumab plus axitinib at our center (Fig. 5a, b). SPI1 expression levels were higher in non-responders (progressive disease or stable disease) than responders (complete disease or partial disease). We obtained 129 ccRCC patients data from three prospective clinical trials of the anti-PD-1 antibody nivolumab[14; 15; 16]. We found that patients with lower *SPI1* expression were more likely to had clinical benefit Additional file 4: Fig. S3). Therefore, *SPI1* may be a marker of immune cells associated with immune infiltration and immunotherapy efficacy in ccRCC.

**Table 3:** The correlations between *SPI1* and gene markers of immune cells in patients with ccRCC

Description	Gene markers	None		Purity	
		Cor	*P* Value	Cor	*P* Value
CD8 + T cell	CD8A	0.574	0.0000	0.525	0.0000
	CD8B	0.583	0.0000	0.536	0.0000
T cell (general)	CD3D	0.663	0.0000	0.618	0.0000
	CD3E	0.668	0.0000	0.625	0.0000
	CD2	0.671	0.0000	0.627	0.0000
B cell	CD19	0.482	0.0000	0.430	0.0000
	CD79A	0.518	0.0000	0.461	0.0000
Monocyte	CD86	0.811	0.0000	0.789	0.0000
	CD115(CSF1R)	0.805	0.0000	0.783	0.0000
TAM	CD68	0.595	0.0000	0.589	0.0000
	IL10	0.540	0.0000	0.490	0.0000
M1 Macrophage	INOS(NOS2)	0.010	0.8229	− 0.045	0.3311
M2 Macrophage	CD163	0.551	0.0000	0.519	0.0000
	VSIG4	0.698	0.0000	0.668	0.0000
	MS4A4A	0.608	0.0000	0.569	0.0000
Neutrophils	CD66b(CEACAM8)	− 0.007	0.8724	0.014	0.7609
	CD11b(ITGAM)	0.743	0.0000	0.723	0.0000
	CCR7	0.563	0.0000	0.515	0.0000
NK	KIR2DL1	0.067	0.1528	0.038	0.4144
	KIR2DL3	0.137	0.0032	0.127	0.0063
	KIR2DL4	0.295	0.0000	0.264	0.0000
	KIR3DL1	0.115	0.0135	0.108	0.0199
	KIR3DL2	0.205	0.0000	0.192	0.0000
	KIR3DL3	0.106	0.0231	0.093	0.0469
	KIR2DS4	0.108	0.0208	0.087	0.0619
Dendritic cell	HLA-DPB1	0.786	0.0000	0.763	0.0000
	HLA-DQB1	0.477	0.0000	0.441	0.0000
	HLA-DRA	0.738	0.0000	0.708	0.0000
	HLA-DPA1	0.695	0.0000	0.658	0.0000
	BDCA-1(CD1C)	0.345	0.0000	0.291	0.0000
	BDCA-4(NRP1)	− 0.035	0.4578	− 0.083	0.0742
	CD11c	0.671	0.000	0.675	0.000
Th1	T-bet (TBX21)	0.425	0.0000	0.384	0.0000
	STAT4	0.441	0.0000	0.388	0.0000
	STAT1	0.523	0.0000	0.476	0.0000
	IFN-γ(IFNG)	0.557	0.0000	0.504	0.0000
	TNF-α(TNF)	0.496	0.0000	0.471	0.0000
Th2	GATA3	0.271	0.0000	0.208	0.0000
	STAT6	0.090	0.0533	0.118	0.0113
	STAT5A	0.655	0.0000	0.613	0.0000
	IL13	0.037	0.4338	0.035	0.4514
Tfh	BCL6	0.012	0.8012	0.016	0.7308
	IL21	0.236	0.0000	0.197	0.0000
Th17	STAT3	0.152	0.0011	0.102	0.0289
	IL17A	0.045	0.3343	0.024	0.6131
Treg	FOXP3	0.647	0.0000	0.607	0.0000
	CCR8	0.549	0.0000	0.495	0.0000
	TGFβ(TGFB1)	0.177	0.0001	0.126	0.0068
T cell exhaustion	PD-1(PDCD1)	0.639	0.0000	0.605	0.0000
	CTLA4	0.573	0.0000	0.530	0.0000
	LAG3	0.604	0.0000	0.564	0.0000

### Methylation status of SPI1 associated with immune infiltrates is a novel prognostic marker in ccRCC

Unlike the positive relationship between the expression of *SPI1* and lymphocytes, hypermethylation of *SPI1* was negatively correlated with lymphocytes in the TISIDB database (Additional file 2: Fig. S2a). Similarly, the methylation status of *SPI1* correlated negatively with both immunoinhibitors, such as LGALS9, PDCD1, LAG3, and TIGIT, and immunostimulators, such as CD86, CD48, TNFS13B, and TNFRSF8, (Additional file 4: Fig. S2b, c). In addition, a negative relationship was observed between the methylation status of *SPI1* and chemokines and receptors, such as CXCL16, CCL5, CXCR6, and CCR1 (Additional file 4: Fig. S2d). DNA methylation can be a prognostic biomarker for RCC survival [32–34]. The correlation between the methylation status of *SPI1* and survival at CpG sites targeted for *SPI1* in ccRCC was explored in MethSurv. The results showed that four CpG sites, including cg03106245, cg06147863, cg07698783, and cg15982099 in *SPI1*, could be prognostic markers for OS in ccRCC (Additional file 1: Table S1). Therefore, the methylation level of *SPI1* is associated with immune infiltrates and may be a novel prognostic marker for survival in ccRCC.

### SPI1 co-expression network in ccRCC

**Fig. 6:**
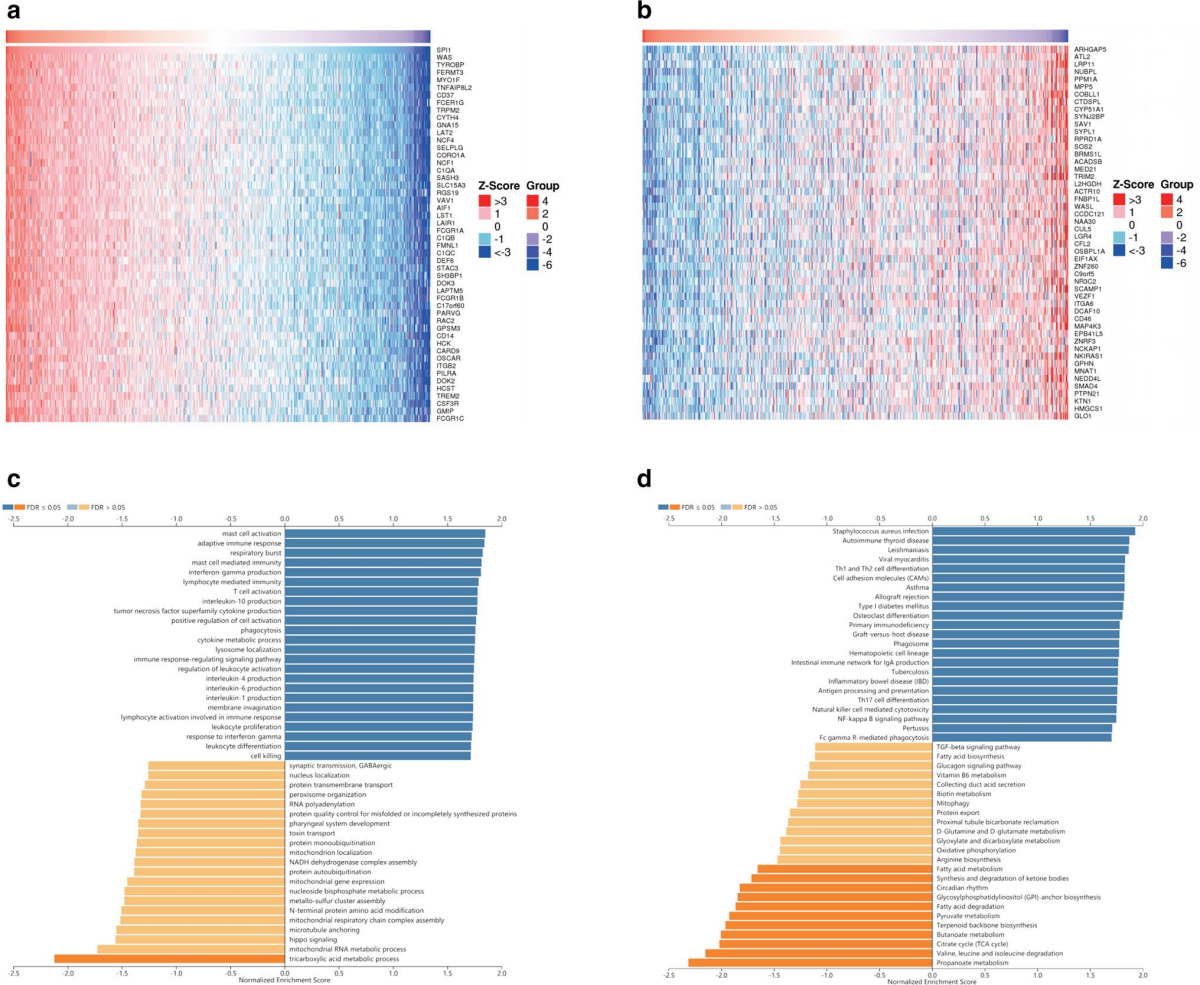
Genes coexpressed with *SPI1* in KIRC using the LinkedOmics database. **a**, **b** The top 50 genes positively and negatively associated with *SPI1* shown in the heat maps. Blue represents negatively linked genes and red represents positively linked genes. **c**, **d** Gene Ontology annotations **c** and Kyoto Encyclopedia of Genes and Genomes pathways (**d**) of *SPI1* in KIRC cohort. KIRC, kidney renal clear cell carcinoma

To predict the biological function of *SPI1*, we performed gene co-expression network analysis of *SPI1* using the LinkedOmics database [30]. We identified 8,264 and 5,265 positively and negatively correlated genes, respectively (p < 0.05) (Additional file 2: Table S2). The top 50 genes that were positively and negatively correlated with *SPI1* expression are shown in the heat maps, respectively (Fig. 6a, b). To clarify the biological functions of genes co-expressed with *SPI1*, we conducted Gene Ontology enrichment analysis using Gene Set Enrichment Analysis. We found that these genes were linked to mast cell activation, adaptive immune response, T cell activation, immune response-regulating signaling pathways, and other immune processes (Fig. 6c). We also performed Kyoto Encyclopaedia of Genes and Genomes pathway analysis to evaluate the functional enrichment of the co-expressed *SPI1* genes and observed enrichment in various immune processes (Fig. 6d). Thus, the *SPI1* expression network is associated with immune and inflammatory responses in ccRCC.

## Discussion

*SPI1* dysregulation has been reported in many cancers [10]. *SPI1* can be a prognostic marker and immunotherapeutic target for gastric cancer patients [35]. Li et al. found that *SPI1* is associated with immune infiltration during oncogenesis [13]. However, the role of *SPI1* in ccRCC is unknown, and the relationship between *SPI1* and immune infiltrates in ccRCC remains unclear. Thus, we comprehensively examined the clinical role of *SPI1* and relationship between *SPI1* and immune infiltrates in ccRCC using our cohort and open-access databases.

In this study, high SPI1 expression level predicted poor OS and PFS in ccRCC patients (Tables 1 and 2). There are several explanations for the poor prognosis of patients with high SPI1 expression level. First, *SPI1* plays a crucial role in myeloid cells and T cells development. A strong positive relationship between *SPI1* expression and macrophages, CD8 + T cells, and CD4 + T cells infiltration was validated via correlation analysis in 183 ccRCC patients. Indeed, macrophages and CD4 + T cells infiltration correlates with poor prognosis in RCC, and our results are consistent with these findings. However, CD8 + T cells infiltration in RCC did not affect clinical outcomes (data not shown). Second, TILs are dominated by M2 macrophages, which represent a major fraction of macrophages in the tumor-associated microenvironment [36]. The TME with high SPI1 expression level can simultaneously maintain high CD68 + macrophages, implying that *SPI1* may be located in M2 macrophages and contributes to tumor-associated macrophage-mediated cancer progression (Fig. 3 and Table 3). *SPI1* expression was positively and strongly associated with T cell exhaustion markers (LAG3 and PDCD1,), and Treg cell markers (FOXP3 and CCR8) (Table 3), suggesting that high *SPI1* expression level inhibits anti-tumor immunity. Third, *SPI1* expression was strongly associated with dendritic cell markers (HLA-DPB1, HLA-DRA, HLA-DPA1, and CD11c) and neutrophils (CD11b) (Table 3). The infiltration of dendritic cells and neutrophils in tumors leads to angiogenesis and cancer progression. In summary, high expression level of *SPI1* correlates with immune cells infiltration, resulting in an immunosuppressive microenvironment and exhaustion of tumor-specific T cells, leading to poor prognosis.

Although cancer immunotherapy has greatly advanced in recent years, the clinical efficacy of immunotherapy is limited in most patients owning to the immunosuppressive tumor environment [37]. He et al. found that *SPI1* is one of the core genes associated with immunotherapy efficacy in triple-negative breast cancers[38]. We found that patients with higher *SPI1* expression levels who were treated by immunotherapy had minor clinical benefits (Fig. 5a, b, Additional file 4: Fig. S3). SPI1 was associated with monocyte /macrophage, T cell, neutrophil, and myeloid dendritic cell infiltration (Fig. 4 and Table 3). Monocytes /macrophages, tumor-infiltrating Tregs, and MDSCs that accumulates in TAM weaken the therapeutic efficacy of immunotherapy [39–42]. This may explain why high *SPI1* expression level is associated with poor immunotherapy efficacy.

Genes in the same pathway or biological process tend to be coexpressed. Co expression and functional enrichment analyses suggested that *SPI1* expression was positively associated with T cell activation and positive regulation of cell activation (Fig. 6c). This may seem contradictory given that *SPI1* expression is prognostic factor for poor survival. This may be because once inflammation persists and T cell activation continues, T cells show loss of interleukin-2 production, cytokine polyfunctionality, and exhausted proliferative capacity [43, 44]. Furthermore, T cell exhaustion is accompanied by increased expression levels of inhibitory receptors (PDCD1, LAG3, CD244, CD160, and TIGIT) [44]. Thus, the sustained activation of T cells in the TME of ccRCC with high *SPI1* expression level ultimately leads to an immunosuppressive state, which requires further investigation. In addition to T cell activation and positive regulation of cell activation, coexpressed genes of *SPI1* participate in various immune cell responses, suggesting that *SPI1* may be associated with various immune-related processes. Recent studies have revealed the potential cause of this association between *SPI1* expression, immune infiltration, and immune molecules. Localized to the nucleus of immune cells, the *SPI1* transcription factor plays a crucial role in myeloid and T cells development. Thus, high *SPI1* expression level correlates with high infiltration levels of macrophages, CD8 + T cells, and CD4 + T cells. Chemokines such as CCL5, CCL4, CXCL9, and CXCL10 secreted by macrophages can be implicated in the recruitment of CD8 + T cells and can promote immune escape by upregulating immunosuppressive molecules (CTLA4, PD1, and LAG3,) (Table 3). Furthermore, cancer cells can secret immune molecules that are dependent on macrophage-derived chemokines to recruit immune cells into TME [45, 46]. In summary, macrophages and T cells with high *SPI1* expression levels may interact with cancer cells by secreting chemokines and immune molecules.

Our study has some limitations. First, our cohort included a relatively small number of patients who received immunotherapy. Second, basic experiments are required to validate the biological role of *SPI1* and to explore its mechanism in ccRCC immunity.

## Conclusions

*SPI1* is a promising prognostic biomarker for ccRCC associated with poor efficacy of immunotherapy, along with high infiltration of immune cells within the TME.

## Supplementary Information

**Additional file 1: Table S1.** The significant prognostic values of CpG in *SPI1.*

**Additional file 2: Table S2.** Genes associated with SPI1 expression in ccRCC

**Additional file 3: Table S3.** Clinical and immune phenotype data for the CheckMate cohorts.

**Additional file 4: Fig. S1.** DNA methylation levels of *SPI1* in ccRCC. (a). Methylation of *SPI1* were lower in ccRCC bulk tissues than that in normal bulk tissues in the UALCAN Database. (b). Correlation analysis of *SPI1 *mRNA expression with *SPI1* promoter methylation status using the cBioPortal database. (c-e).Promoter methylation level of *SPI1 *in ccRCC tumor bulk tissues of different tumor gade (c), tumor stage (d), and nodal metastasis status(e) using the UALCAN database. ccRCC, clear cell renal cell carcinoma; *, p < 0.05; **, p < 0.01; ***, p < 0.001; ns, non-significant. **Fig. S2.** Immune infiltrates in ccRCC are associated with the methylation status of *SPI1*. (a) Relationships between the methylation status of *SPI1 *and activated CD4 T cells, activated CD8 T cells, and activated dendritic cells in patients with ccRCC using the TISIDB database. (b-d) Relationships between the methylation status of *SPI1* and immunostimulators (b), immunoinhibitors (c), and chemokines/receptors (d) in patients with using the TISIDB database. KIRC, kidney renal clear cell carcinoma; ACT_CD4, activated CD4+ T cells; ACT_CD8, activated CD8+ T cells; ACT_DC, activated dendritic cells. **Fig. S3.** Relationship between *SPI1* expression and clinical benefit of immunotherapy in prospective clinical trials in ccRCC. (a) Patients with lower* SPI1* expression benefit more from immunotherapy. CB, clinical benefit; NCB, no clinical benefit; ICB, intermediate clinical benefit.

## Data Availability

The datasets analyzed for this study can be found in the TIMER database (https://cistrome.shinyapps.io/timer/), the UALCAN database (http://ualcan.path.uab.edu/), the TISIDB database (http://cis.hku.hk/TISIDB), the cBioPortal database (www.cbioportal.org), and the LinkedOmics database (http://www.linkedomics.org/login.php). The raw data from our central supporting the conclusions of this article will be made available by the authors, without undue reservation.
