# Supplementary material for: SPI1 is a prognostic biomarker of immune infiltration and immunotherapy efficacy in clear cell renal cell carcinoma
Source: Discov Oncol. 2022 Dec 7;13:134. doi: 10.1007/s12672-022-00592-0 (PMC9729685; doi:10.1007/s12672-022-00592-0)
Supplement: Supplementary file 1 — Additional file 1: Table S1. The significant prognostic values of CpG in SPI1. [file 12672_2022_592_MOESM1_ESM.docx]

| Name | HR | CI | LR_test_  pvalue | UCSC_RefGene  _Group | Relation_to_UCSC  _CpG_Island |
| --- | --- | --- | --- | --- | --- |
| cg01539849 | 1.545 | (0.956;2.495) | 0.064 | 3'UTR | Island |
| cg03106245 | 0.541 | (0.329;0.891) | 0.010 | 5'UTR;1stExon | Island |
| cg03301240 | 0.703 | (0.479;1.031) | 0.072 | TSS1500 | S_Shore |
| cg03565868 | 0.704 | (0.463;1.072) | 0.111 | TSS200 | S_Shore |
| cg06147863 | 0.605 | (0.368;0.995) | 0.037 | 5'UTR;1stExon | S_Shore |
| cg06784824 | 1.606 | (0.977;2.64) | 0.050 | Body | Island |
| cg07675031 | 0.748 | (0.509;1.099) | 0.138 | 1stExon | Island |
| cg07698783 | 0.495 | (0.29;0.843) | 0.005 | Body | N_Shelf |
| cg10435245 | 0.721 | (0.491;1.061) | 0.097 | 5'UTR;1stExon | Island |
| cg14088811 | 0.811 | (0.552;1.19) | 0.284 | 5'UTR;1stExon | Island |
| cg15982099 | 0.547 | (0.332;0.9) | 0.012 | Body | Island |
| cg16517172 | 0.654 | (0.405;1.056) | 0.071 | TSS200 | S_Shore |

**Supplementary table 1** The significant prognostic values of CpG in *SPI1*.
