# Supplementary material for: SPI1 is a prognostic biomarker of immune infiltration and immunotherapy efficacy in clear cell renal cell carcinoma
Source: Discov Oncol. 2022 Dec 7;13:134. doi: 10.1007/s12672-022-00592-0 (PMC9729685; doi:10.1007/s12672-022-00592-0)
Supplement: Supplementary file 4 — Additional file 4: Fig. S1. DNA methylation levels of SPI1 in ccRCC. (a). Methylation of SPI1 were lower in ccRCC bulk tissues than that in normal bulk tissues in the UALCAN Database. (b). Correlation analysis of SPI1 mRNA expression with SPI1 promoter methylation status using the cBioPortal database. (c-e).Promoter methylation level of SPI1 in ccRCC tumor bulk tissues of different tumor gade (c), tumor stage (d), and nodal metastasis status(e) using the UALCAN database. ccRCC, clear cell renal cell carcinoma; *, p < 0.05; **, p < 0.01; ***, p < 0.001; ns, non-significant. Fig. S2. Immune infiltrates in ccRCC are associated with the methylation status of SPI1. (a) Relationships between the methylation status of SPI1 and activated CD4 T cells, activated CD8 T cells, and activated dendritic cells in patients with ccRCC using the TISIDB database. (b-d) Relationships between the methylation status of SPI1 and immunostimulators (b), immunoinhibitors (c), and chemokines/receptors (d) in patients with using the TISIDB database. KIRC, kidney renal clear cell carcinoma; ACT_CD4, activated CD4+ T cells; ACT_CD8, activated CD8+ T cells; ACT_DC, activated dendritic cells. Fig. S3. Relationship between SPI1 expression and clinical benefit of immunotherapy in prospective clinical trials in ccRCC. (a) Patients with lower SPI1 expression benefit more from immunotherapy. CB, clinical benefit; NCB, no clinical benefit; ICB, intermediate clinical benefit. [file 12672_2022_592_MOESM4_ESM.docx]

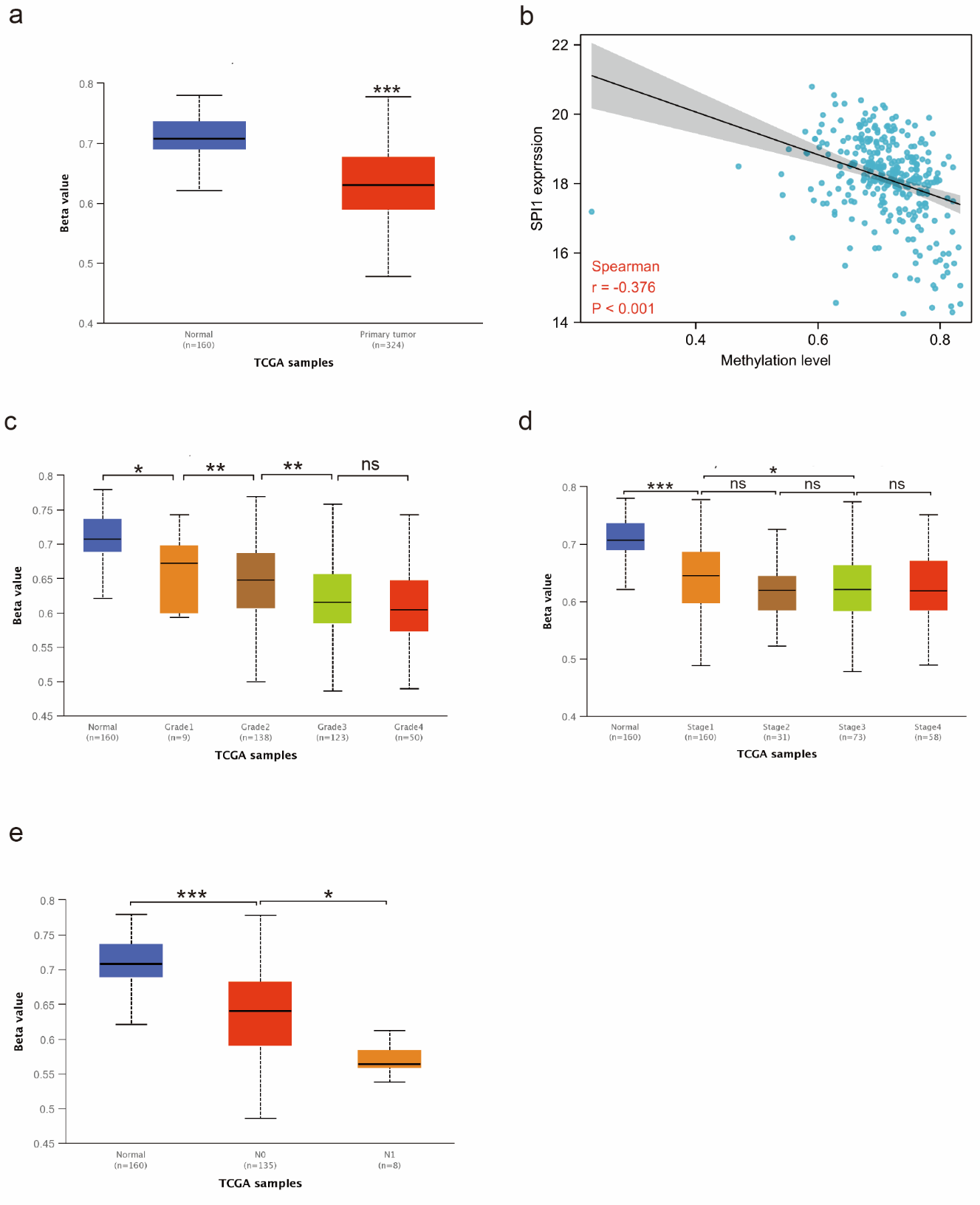


**Supplementary fig. 1** DNA methylation levels of *SPI1* in ccRCC

(a). Methylation of *SPI1* were lower in ccRCC bulk tissues than that in normal bulk tissues in the UALCAN Database. (b). Correlation analysis of *SPI1* mRNA expression with *SPI1* promoter methylation status using the cBioPortal database. (c-e).Promoter methylation level of *SPI1* in ccRCC tumor bulk tissues of different tumor gade (c), tumor stage (d), and nodal metastasis status(e) using the UALCAN database. ccRCC, clear cell renal cell carcinoma; *, p < 0.05; **, p < 0.01; ***, p < 0.001; ns, non-significant


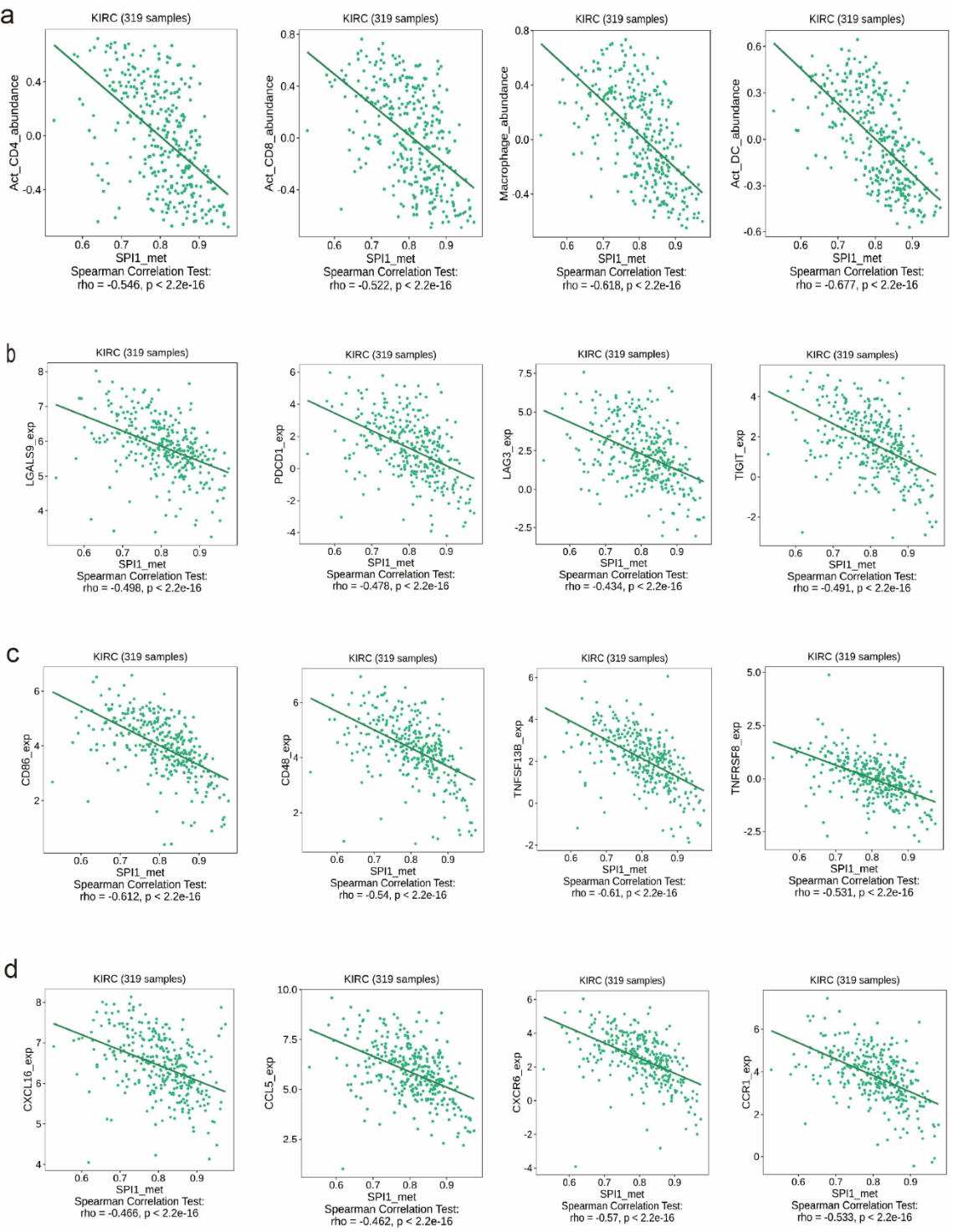


**Supplementary fig. 2** Immune infiltrates in ccRCC are associated with the methylation status of *SPI1*

(a) Relationships between the methylation status of *SPI1* and activated CD4 T cells, activated CD8 T cells, and activated dendritic cells in patients with ccRCC using the TISIDB database. (b-d) Relationships between the methylation status of *SPI1* and immunostimulators (b), immunoinhibitors (c), and chemokines/receptors (d) in patients with using the TISIDB database. KIRC, kidney renal clear cell carcinoma; ACT_CD4, activated CD4+ T cells; ACT_CD8, activated CD8+ T cells; ACT_DC, activated dendritic cells.


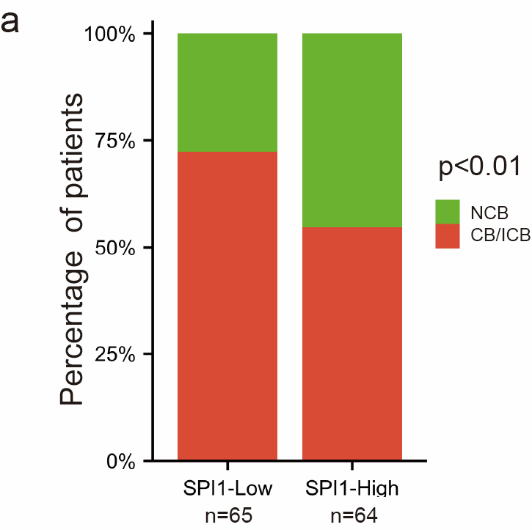


**Supplementary Fig 3** Relationship between *SPI1* expression and clinical benefit of immunotherapy in prospective clinical trials in ccRCC.

(a) Patients with lower *SPI1* expression benefit more from immunotherapy. CB, clinical benefit; NCB, no clinical benefit; ICB, intermediate clinical benefit.
